# Fabrication of large size graphene and Ti- MWCNTs/ large size graphene composites: their photocatalytic properties and potential application

**DOI:** 10.1038/srep14242

**Published:** 2015-09-18

**Authors:** Kefayat Ullah, Won-Chun Oh

**Affiliations:** 1 Department of Advanced Materials Science & Engineering, Hanseo University, Seosan-si, Chungnam-do, Korea, 356-706

## Abstract

Large size graphene (LSG) and multiwall carbon nanotubes (MWCNTs) on LSG were synthesized on a copper surface via chemical vapor deposition (CVD) at low temperature and normal pressure. The LSG were formed through an easy chemical cyclic reaction in which liquid benzene was heated to a temperature below its boiling point to create benzene vapors as graphene precursor material. The reaction mechanism was observed, and the time-dependent analysis of the reaction revealed that mounds of the carbon nanotubes had grown as a result of the island that was found on the LSG sheet. The implications of the mechanism that we have introduced were investigated by coating a titanium sheet on the MWCNTs/LSG and LSG on the semiconductor electronic device. The photonic response was observed to be markedly high, which can be attributed to the positive synergetic effect between the Ti and LSG sheet of our prepared composites.

Graphene consists of a single layer of carbon atoms that can be obtained from highly-oriented pyrolytic graphite through mechanical exfoliation, and it has been shown to have outstanding electronic, optical, and mechanical properties. Although various techniques have been developed to produce graphene on a solid material substrate^1^,^2^,^3^,^4^,^5^, chemical vapor deposition (CVD) has been considered to be the route with the highest potential to synthesize large area graphene. The highly-oriented, polycrystalline structure with a maximum density of grains and domains areas in CVD-grown graphene is the reason for which devices are produced with varying optical and electrical properties, and these variations are the main issue that is yet to be addressed in order to obtain graphene-based nano electronics devices. Graphene has been obtained in limited sizes so for because the films are mostly produced through the exfoliation of natural graphite, and graphite oxidation is not a scalable method^6^,^7^,^8^,^9^,^10^. It is therefore still a challenge to produce large size graphene in order to fulfill the requirements to produce optoelectronic and photonic devices based on graphene. Another method that has been introduced involves the desorption of Si from single-crystal SiC, which results in a multilayered and monolayer graphene structure^11^,^12^. The main advantage of the epitaxial technique that monolayer graphene can be produced in a controllable manner. However, this method has numerous disadvantages, including the need to cover atomically smooth surface with a single layer sheet of graphene during synthesis. Therefore, advances are needed in SiC substrate preparation and pit knowledge of graphene nucleation and growth.

CVD is a relatively simple technique that can be used to obtain graphene with the desired features. CVD involves the decomposition of carbon precursor materials, that is, hydrocarbons or polymeric components, by adding heat and some metallic catalysts, such as Ni, Pt, Cu, etc., that have been found to improve the growth of graphene. The optimum conditions to produce large size graphene through CVD have yet to be determined, and therefore, various approaches have been proposed^13^. Graphene has been developed on several different metals. For example, growth on nickel metal produces graphene with a small grain size and multilayer sheets have been found on grain boundaries^14^,^15^. Recently, graphene nano ribbons have attracted an increasing amount of research interest due to their extraordinary electronic and optoelectronic properties. Tunneling microscopy and scanning tunneling microscopy techniques have been used to study the edge effects in large area graphene, and the altered electronic and optical properties have been reported to clearly explain the effect of the edge, grain size and growth area of graphene. Similarly, Peng *et al.* studied the growth of epitaxial graphene (EG) on insulating silicon carbide (SiC) surfaces by developing a promising route to produce large-scale high-quality graphene. The amorphous carbide precursor has been transformed to graphene nanosheets through chlorination at low temperature and ambient pressure, and some exceptional outcomes have been obtained. These results suggest the importance of the size effect when synthesizing CVD grown graphene on metal substrates^16^,^17^,^18^,^19^,^20^.

In this study, we investigate a novel CVD growth process for LSG/MWCNTs on copper foil at low temperature of 500 °C. This procedure consists of two steps. First, we observe a large size micrometer graphene sheet on Cu foil at the appropriate temperature, and in the second step, we develop the growth mechanism for LSG and MWCNTs/LSG at very low temperature. We thus suggest a novel growth mechanism for LSG and MWCNTs/LSG through chemical vapor deposition on a copper foil at low temperature. The novel mass production of LSG and MWCNTs/LSG on Cu foil may be very useful in the fabrication of ultrafast charge transfer for next generation optical and electronic devices. Finally, we suggest a potential application by coating Ti on the MWCNTs/LSG on Cu foil and coating LSG on electronic devices.

## Results

### Morphological formation and growth of LSG and MWCNTs/LSG

**Figure 1:**
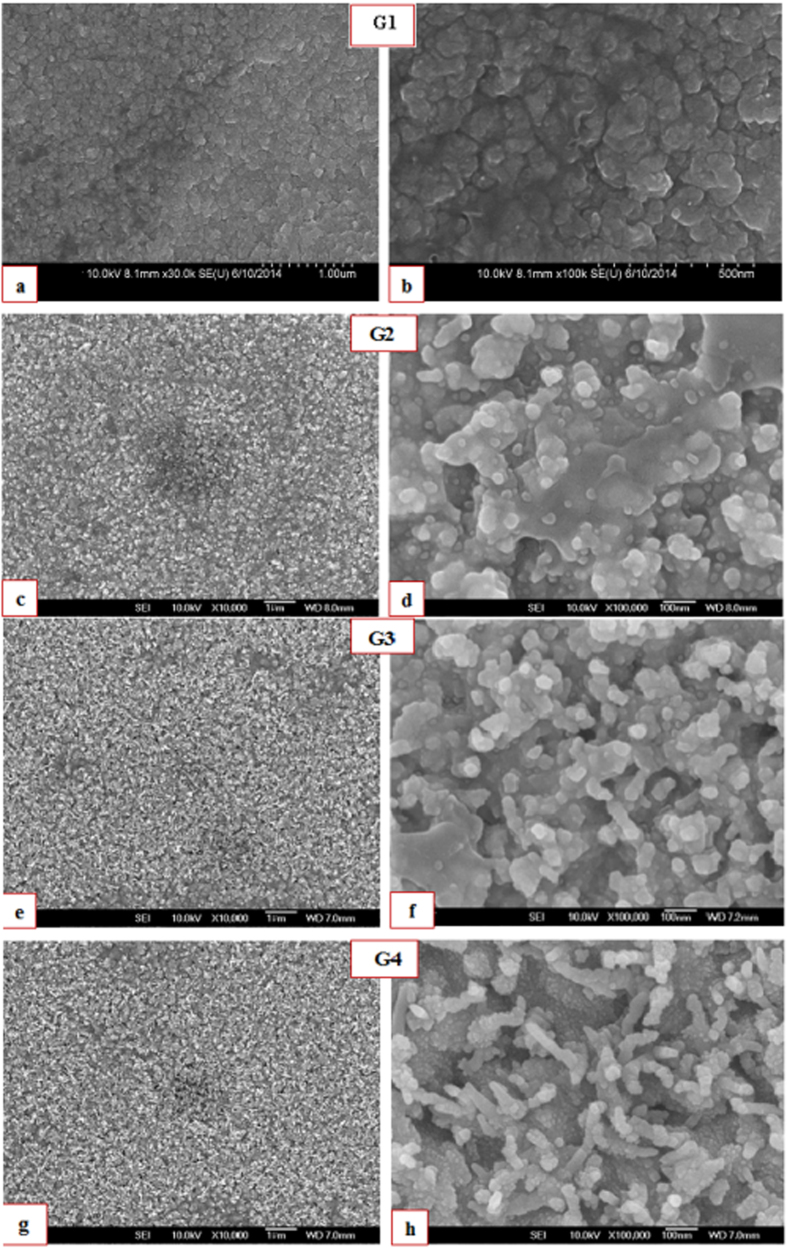
(**a**–**h**) FESEM images of the as-prepared LSG and MWCNTs/LSG (**a**,**b**) G1, 1 minute reaction time (**c**,**d**) G2, 2.5 minute reaction time (**e**,**f**) G3, 4 minute reaction time (**g**,**h**) G4, 5 minute reaction time.

FESEM images of the as-prepared CVD-grown LSG and MWCNTs/LSG assembly are shown in Fig. 1(a–h). It is clear that the quantity of large area graphene increases as the reaction time decreases. Figure 1(a,b) describes the FESEM image of the G1 sample with a 1 minute reaction time. In this figure, the G1 sample provides a vibrant picture of the morphology of graphene. The graphene sheets mainly appear as multilayers that are separated by pores that formed on the Cu substrate. These pores can be considered to have supported the reaction spaces to attach the nanoparticles for device applications. The image shows that no other spot or mound could be found on the surface of graphene, and this provides us a clue that a 1-minute reaction time is suitable for the LSG layer to grow on the copper surface at 500 °C.

When the reaction time increases to 2 and half minutes by keeping the temperature at 500 °C, a small dot appears on the surface of the LSG, and these dots are referred to as carbon nanotube seeds, as shown in Fig. 1(c,d). The sample in Fig. 1(c,d) with 2.5 minute reaction time is labeled as G2. In this image, small dots can be very clearly seen to be spotted white in color. These dots are further responsible for the concentric graphene sheet of the CNTs rolling into a tube format. A further increase of the reaction time to 4 minutes results in the dot found in the G2 sample to be extended linearly, and rod-type MWCNTs can be seen on the LSG surface. The rod type shape can be clearly seen in Fig. 1(e,f) in the sample labeled G3. Figure 1(g,h) shows the G4 sample with 5 minutes of reaction time. The MWCNTs on the Cu substrate are clearly visible in the tube-type shape, and the density of the MWCNTs is quite large, so the Cu grains are not visible in this image. The G4 sample shows that when the reaction time exceeds 2 minutes, the CNT seeds grown on the surface of the graphene sheet. The large numbers of MWCNTs were wrinkled and curled around each other, so the width of the MWCNTs also increased relative to those in the G3 sample. These MWCNTs that formed are found to be vertical on LSG sheet, as confirmed in the AFM images. Our observations further show that by controlling a number of experimental parameters, these MWCNTs can be conveniently grown on the graphene sheet, which may be beneficial for the production of future graphene-based nano electronics devices.

**Figure 2:**
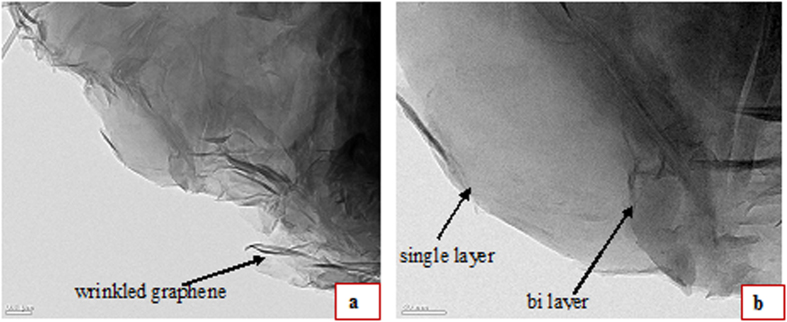
TEM images of the G1 sample (1 minute reaction time) (a) 0.1 μm (b) 50 nm.

The production of large area graphene and the number of layers of the G1 sample were investigated through the TEM image shown in Fig. 2(a,b). Figure 2(a) shows that smooth large area graphene with mostly a single layer was produced. The dark region shows the stacked, mostly bi layer graphene with the presence of some wrinkles. These wrinkles may be associated with the difference in the thermal coefficient between the graphene and the Cu film^21^. The G1 sample produced with a 1-minute reaction time was further evaluated using high-resolution electron microscopy in order to study the detailed structure and the edges of the large area graphene. Figure 2(b) shows a high-quality single-layer graphene sheet without any MWCNTs on the graphene surface. Our results further confirm that the benzene vapors flowed through the CVD at low temperature (500 °C) and normal pressure can produce LSG composites. The LSG sheet in Fig. 2(b) shows the composition of mostly single-layer and bi-layer LSG.

**Figure 3:**
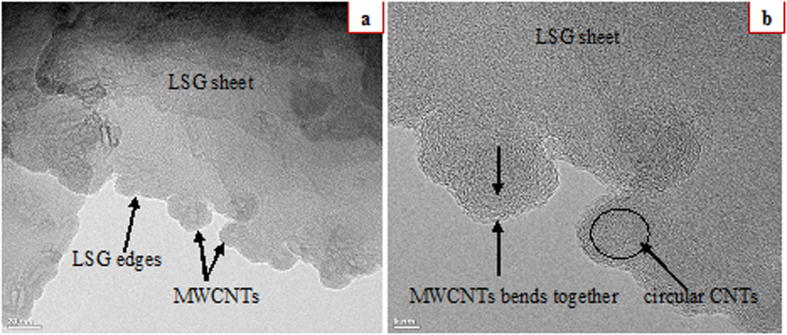
(**a**,**b**) HRTEM images of the MWCNTs/LSG composite; G4 sample with 5 minute reaction time (**a**) 20 nm (**b**) 5 nm.

The production of MWCNTs on the LSG sheet was verified through a transmission electron microscopy (TEM) analysis of the G4 sample. Figure 3(a,b) shows the TEM image of the G4 sample with mount-type MWCNTs. The tip of the MWCNTs is around 15 nm in diameter, and by increasing the reaction time, these small dots were found to spread, forming MWCNTs vertically. A large number of these MWCNTs appear to have gathered around each other in bundles, and the TEM image in Fig. 3(b) show that the circular MWCNTs were wound together in bundle-type shapes. This means that MWCNTs were grown at the edges of our large size graphene, and the density is reduced at the interior of the sheet. These results provide new insight into the MWCNTs/LSG composites. Thus, the edge CNTs may be helpful in developing ultrafast nano electronic devices by providing sharp anchoring sites.

### Physicochemical characterization of the LSG and MWCNTs/LSG

**Figure 4:**
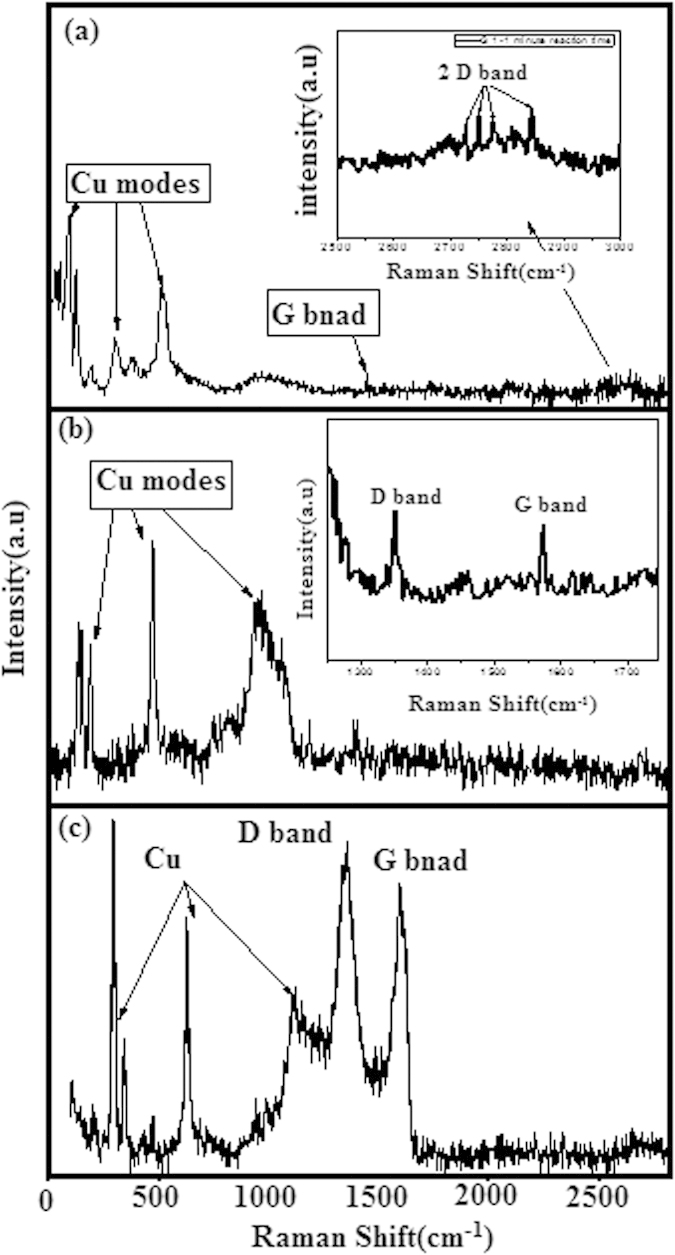
(**a**–**c**) Raman spectra of (**a**) G1 sample with 1 minute reaction time (**b**) G3 sample with 4 minutes reaction time (**c**) G4 sample with 5 minutes reaction time.

Raman spectroscopy is a powerful technique that can be used to study the structure and electronic properties of carbon materials, including graphene/graphite and carbon nanotubes. Figure 4(a) depicts the Raman spectrum of the G1 sample. The temperature of the furnace remained at 500 °C with a reaction time of 1 minute. The Raman signature for D band, G band of carbon-based materials was observed in the CVD-grown LSG. The modes positioned between 80 and 300 cm^−1^ from the excitation line are associated with the Cu present in the sample. Cu arises as a result of scratching the graphene composite from the Cu foil.

Figure 4(a) presents unusual results for the G1 sample rather than the ordinary Raman spectra of the LSG and CNTs. The figure shows a small intensity peak [inset Fig. 4(a)] at around 2700 cm^−1^ that corresponds to the 2D peak in graphene. This 2D peak was further split into four Raman peaks, and the dispersion of the peak yielded information on the number of layers and the stacking order in the graphene system. 2D peaks in bilayer graphene have been reported to be split into four components, which further increases in the layer and bulk area of graphene and leads to lower intensity peaks^22^,^23^. In our case, we believe that the surface area of graphene is very large and that the intensity of the peaks is therefore very low relative to the metallic band in the results of the Raman spectroscopy. Figure 4(b) shows the Raman spectra for the G3 sample with a reaction time that was kept at 4 minutes at 500 °C. From Fig. 4(b), it is clear that the small-intensity G and D bands are present [inset Fig. 4(b)]. Cu peaks were also observed.

The results of our study indicate that by increasing the reaction time, the Raman band intensities of the G band and D band shift towards a lower frequency. This allows the MWCNTs to start growing on the LSG sheet. The other reason for the variation in intensity is the large number of MWCNTs that roll together, encouraging phonon confinement and thus reducing the intensity^24^,^25^. These observations show that the attachment of MWCNTs/LSG on Cu during CVD would probably affect the electronic transition, and hence, the dispersive behavior of the signature bands could be observed.

The laser excitation also has an effect on the Raman band shift and the variation in intensity. In the MWCNTs, the bands could be attributed to the Raman resonance condition, and the incident laser on the carbon nanotubes can yield an optical resonance profile for a given mode at a vibrating frequency of the respective modes^26^,^27^,^28^,^29^. In Fig. 4(b), the G band frequency could be seen at 1592 cm^−1^ while the D band frequency was observed at 1352 cm^−1^. For the MWCNTs, the large number of CNTs with different diameters affects the characteristic G band, and therefore theses are found close to the G band frequency of graphite^30^. The peaks between 300 and 500 cm^−1^ were attributed to the Cu that was used as a substrate^31^,^32^,^33^.

Figure 4(c) shows the Raman spectrum of the G4 sample with 5 minutes of reaction time. The observation of Fig. 4(c) shows that with an increasing reaction time, the D band appears in the spectrum. A defect band is observed unlike in other samples, and this may be due to the large number of MWCNTs that were grown on the LSG sheet. The morphology of the G4 sample observed in the FESEM and TEM images makes it quite clear that the increase in reaction time results in an increase in the density of the MWCNTs due to the formation of CuO on the surface, which increases the amount of grains and thus provides a higher number of reaction sites to grow the CNTs. This produces a high surface energy that provides more active sites for MWCNTs on the surface of the material^34^,^35^,^36^.

**Figure 5:**
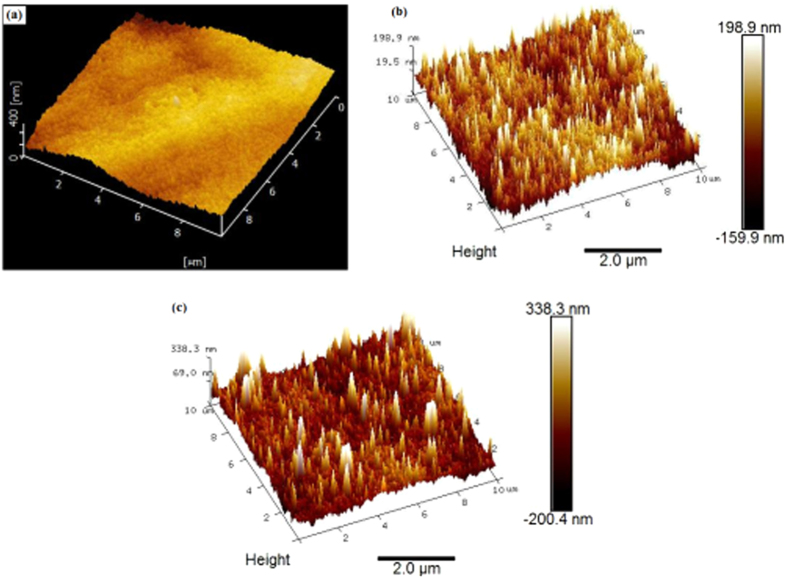
(**a**–**c**) AFM images of LSG and MWCNTs/LSG (**a**) G1 (**b**) G3 (**c**) G4.

We then used AFM topography images to determine the thickness and the height of the LSG and MWCNTs/LSG composites. The LSG and MWCNTs/LSG composites are shown in Fig. 5(a–c). The G1 sample in Fig. 5(a) gives larger area graphene with some dark regions that are considered to be valleys between the two graphene domains. The depths of these valleys is observed to be about 350 to 400 nm to minimize the graphene domains, and these graphene edges may be very useful to create Li-ion batteries that possess a remarkable storage capacity, as reported elsewhere^37^. Figure 5(b,c) shows a comparison of the MWCNTs/LSG composites obtained with different reaction times. Figure 5(b) shows the G3 sample, which was produced with 4 minutes of reaction time, and Fig. 5(c) shows the G4 sample produced with 5 minutes of reaction time. These figures show obvious differences between the heights of the MWCNTs as a result of the variation in the reaction times. Figure 5(b) shows the G3 sample with MWCNTs that have a height of around 198 nm, and Fig. 5(c) shows the G4 sample where this height was measured at around 338 nm. The AFM height images of the MWCNTs clearly show that the CNTs gathered in a rolled-type shape with a sharp tip and close ends. The growth of the CNTs shown in Fig. 5(b,c) could be consistently achieved by using a large area substrate material.

The surface growth of vertically-aligned CNTs on the substrate material at a micrometer scale with nonmetric effect is very important for next generation devices. The AFM images revealed the surface construction of MWCNTs leading towards nanohill formation of carbon nanotubes, which is unique, and this formation is a complex phenomenon that may be the result of the different competing factors, such as temperature, reducing atmosphere, oxide reduction during the CVD process, and benzene decomposition. This elusive, complex phenomenon will require focused investigation in the future to explore the outstanding physical and chemical properties of the MWCNTs/LSG composites.

## Discussion

The Supplementary Fig. S1 shows the system that was specially designed to produce our LSG and MWCNTs/LSG. A detailed description is given in a later section. The LSG film was produced directly on the Cu foil by using a surface-catalyzed process for mass production. The film that was obtained consists predominantly of graphene with a multilayer structure. However, a single-layer and bilayer graphene are usually found to have a larger size. The advantage of using our experimental process is that it can be used to produce the LSG and MWCNTs/LSG on the Cu foil at a very low temperature to achieve advanced nano electronics technology by controlling additional parameters.

**Figure 6:**
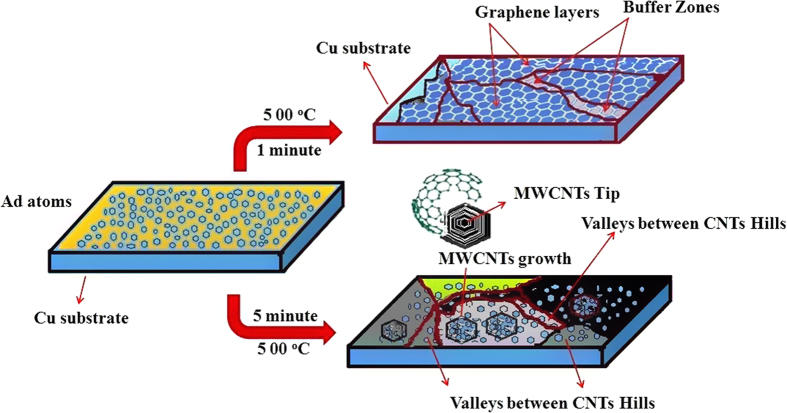
Schematic drawings of the mechanism to form the LSG and MWCNTs/LSG composites.

The scheme to form the LSG and MWCNTs is depicted in Fig. 6. As the schematic diagram shows, the formation of LSG and MWCNTs has been achieved by varying the reaction time. Due to assimilated nature of the synthesis method that is introduced, the resulting carbon hybrid structure shows an interconnected structure and robust contact between the carbon nanotubes and the large area graphene.

Supplementary Fig. S2 (a,b) shows the mechanism for the formation with a schematic diagram. The AFM images in Supplementary Fig. S2(a) for the 5-minute reaction G4 sample further confirms that valleys are found and are surrounded by MWCNTs with small hill-type structures. The valleys can be referred to as buffer zones, and these buffer zones are extended when the reaction time increase up to 5 minutes and the small dots are converted to MWCNTs. The LSG in Supplementary Fig. S2 (b) obtained with a 1-minute reaction time shows that a different region with exchanged morphology can be observed. These regions can be attributed to gross sites to form MWCNTs when the temperature increases up to 5 minutes.

**Figure 7:**
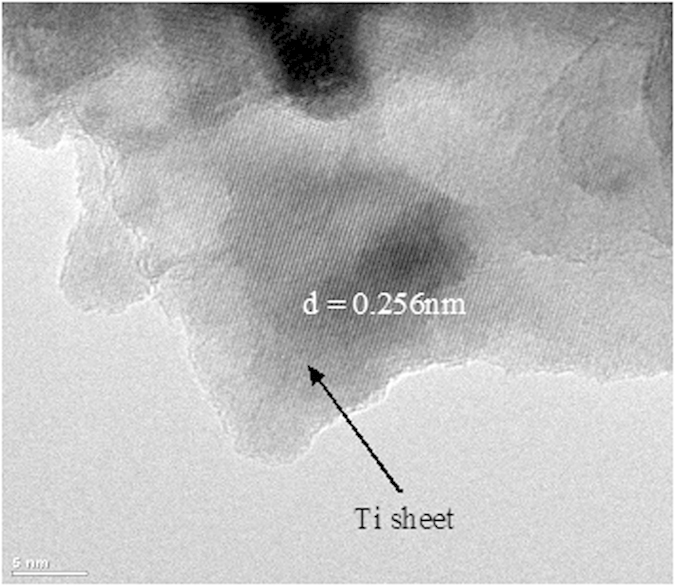
HRTEM image of Ti-coated LSG/MWCNTs composite at 500 °C.

The samples were coated by titanium through the same process that was explained above by using TNB as a precursor material. During the CVD process, we observed that the Ti attached as a sheet similar to graphene that covered the maximum surface of the graphene samples. Supplementary Fig. S3 presents the XRD patterns of the MWCNTs/LSG coated in Ti by keeping the temperature at 500 °C. The diffraction peak of Ti (100) has a very strong intensity that tends to suppress the (002) peak of graphene. The Ti sheet exhibits the characteristic (100), (002), (110), (200), (112) reflections that correspond to a crystal phase (JCPDS PDF#: 00-065-3362). After the titanium precursor material is introduced on the G4 sample, we find that the titanium sheet is attached to the graphene and covers the entire surface. The HRTEM image of the G4 sample coated with Ti in a sheet-like format is depicted in Fig. 7.

**Figure 8:**
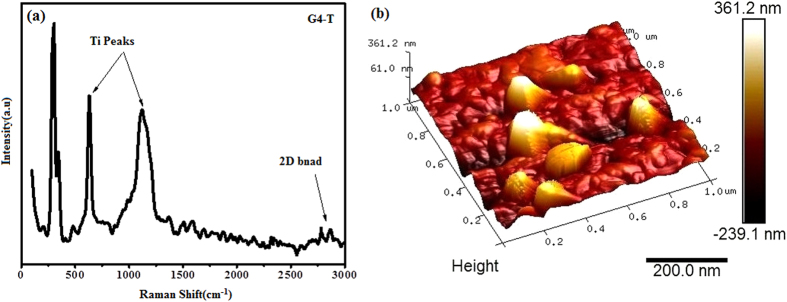
(**a**,**b**) Ti coated G4 sample (**a**) Raman spectra (**b**) AFM images.

The Raman spectrum of the Ti coated G4 sample is shown in Fig. 8(a). This figure clearly shows that the Ti species were easily attached to the surface of the MWCNTs/LSG composites. In Fig. 8 (a) the Raman peaks that are associated with specific Ti species are observed at 630, 530 and 1116 cm^−1^. The peak at around 1100 cm^−1^ can be attributed to the Ti lattice t_d_ symmetry^38^. These results also suggest that large intensity peaks suppress the Raman band intensities of graphene. Figure 8(b) describes the AFM image of the Ti coated MWCNTs/LSG composites. The AFM images of the topography show that the entire surface of the composite has been mostly covered by the Ti sheet. There are small dark regions in the image, and these regions are considered to be the valleys that were found in the FESEM images of the G4 sample while the mounds may be attributed to MWCNTs covered by the Ti sheet.

The photo-catalytic properties of the LSG/MWCNTs and Ti-MWCNTs/LSG that were obtained can be examined by using Rh.B degradation under visible light. The scratch sample from the copper foil was used as powder for the photocatalytic experiment. The Rh.B photodegradation rate was assessed under visible light (8W, λ > 420 nm, KLD-08L LED lamp). The photo activities of the LSG, MWCNTs/LSG and Ti-MWCNTs/LSG were determined according to the degradation rate of Rh.B as a function of time under visible irradiation. In order to attain adsorption/desorption equilibrium, the solution was kept in the dark for 2 h. After adsorption/desorption equilibrium had been achieved, the solution was irradiated with visible light for 30 minutes. For the analysis, the sample was periodically withdrawn, and the solids were removed via centrifugation.

The dye concentration of the sample was then analyzed using a UV-visible spectrometer. Under visible light, the energy state of the electrons changed from a valance band to a conduction band, and the excited electrons reacted with the adsorbed oxygen, resulting in the formation of free radicals. These free radicals reacted with the dye, resulting in the mineralization of the dye into simple molecules. During the experiments, a decrease in the color intensity of the solution was gradually observed over time due to the adsorption and degradation of Rh.B. The Rh.B concentration in the solution was determined to be a function of the irradiation time from the change in absorbance at a wavelength of 554 nm. The results of the photo degradation (Optical absorption spectra) are shown in the Supplementary Fig. S4(a–c). Supplementary Fig. S4(d) also shows the change in the concentration of the Rh.B with different irradiation times in the presence of the photocatalysts that were prepared. The general degradation efficiency of Rh.B with all samples under visible light followed the order of Ti-MWCNTs/LSG > MWCNTs/LSG > LSG. 80% of the Rh.B can be seen to have been degraded by the Ti-MWCNTs/LSG nanocomposite. The Rh.B adsorption was slightly reduced with the G1 and G4 samples, and the graphene coupled with the Ti supported by the MWCNTs shows an improved catalytic activity as a result of the high charge separation that was induced by the synergetic effects of graphene and Ti while the MWCNTs provide a high charge transfer path between the LSG and Ti. Thus, the photocatalytic properties of the Ti-MWCNTs/LSG composites are enhanced^39^.

Kinetic studies were performed on the basis of the degradation rate of the organic dye, and the reactions between the dye molecules and the catalyst materials can be expressed according to the Langmuir- Hinshelwood model, as shown in Supplementary Fig. S5(a), which is explained in detail in our previous report^40^. Supplementary Fig. S5 (a) shows that the slope of the linear plots should be equal to the apparent first order rate constant (K_app_). The K_app_ values provide the degradation rate of the Rh.B molecules by the photocatalyst materials under the influence of visible light. The values of the K_app_ rate constant are listed in Supplementary Fig. S5(a), and the Rh.B degradation rate constant for the Ti-MWCNTs/LSG composite was found to be 7.15 × 10^−3^ min^−1^ under visible light, which was much higher than the G1 and G4 sample in our nanocomposites. These results further confirm that the Ti-MWCNTs/LSG nanocomposites are a much more effective catalyst material than the other nanocomposites in the CVD-grown LSG/MWCNTs nanocomposites.

The photocatalytic properties of the CNT composites with other semiconductor materials have been extensively studied over the last two decades, and the effect of the CNTs has been made quite clear from these investigations. For example, Lei *et al.* synthesized CNTs-Fe/Ni-TiO_2_ composites, and an improvement was observed in the catalytic effect due to the role of the CNTs in the composites^41^. Similarly, Zhao *et al.* studied the properties of CNTs-TiO_2_/Al_2_O_3_ membranes in terms of their photocatalytic function and provided a significant description of the use of CNTs-based materials for purification systems^42^. Such research clearly indicates the importance and the high charge transfer properties of the CNTs that support our graphene-based composites system.

To further demonstrate the photo-stability and cyclic performance of the Ti-MWCNTs/LSG composite photo-catalysts, cyclic experiments were carried out with Rh.B as an organic dye. Supplementary Fig. S5(b) shows that the photo-catalysts do not exhibit a significant loss in photo-catalytic activity after three runs of Rh.B degradation, which indicates that the Ti-MWCNTs/LSG photo-catalyst has a high stability and cannot be photo-corroded during the photo-catalytic oxidation of the Rh.B molecules. Thus, the Ti-MWCNTs/LSG composite photo-catalyst is promising for practical use in environmental purification. The reusability test of the Ti-MWCNTs/LSG photocatalyst on the organic dyes shows the stability of the catalyst composites. The catalyst that is reused does not show any noticeable changes in its degradation efficiency, which reinforces idea that these catalysts have an excellent chemical stability that is useful in practical applications.

A semiconductor index device was considered for coating with our proposed CVD method. The actual image of the device is shown in Supplementary Fig. S6. After coating the material with CVD, the material was found to be a remarkable heat sink due to the attachment of large area graphene. This shows the importance of using our new method to produce low-temperature CVD graphene for device application. Since graphene/CNTs composites provide numerous benefits, several studies have been devoted to studying these composite nanostructures^43^,^44^,^45^,^46^. In light of the above experimental results, our work can be considered to be a novel, one-step, low-temperature techniques to produce LSG/MWCNTs composites structures. The use of a separator between the Cu foil and the catalyst materials is considered to be very important in reducing the interaction between the Cu and the metal catalyst materials. In our case, the most interesting point is that the LSG provides a plate to grow MWCNTs and also act as a separator between the Cu foil and the MWCNTs.

In summary, we have investigated a new, short route to produce large area graphene. This method can be used to produce large area graphene for industry at bulk by further controlling the experimental parameters to determine the optimum values. We demonstrate the production of large area graphene with a very short reaction time in an argon environment by using chemical vapor deposition. An increase in the reaction time to 5 minutes provides interesting results due to the production of ~350 nm long, vertically-aligned MWCNTs that are homogenously distributed through the copper foil, as shown in the AFM images. The FESEM images show that that the island found on the graphene can further give rise to vertical CNTs. The valleys that are observed between the graphene domains may be a result of the use and handling of a very thin copper foil. The UV-visible spectrum clearly shows an enhanced catalytic effect for the Ti-MWCNTs/LSG nanocomposite under visible light, suggesting an optimal loading effect of graphene to TiO_2_. This high level activity may be a result of the positive synergy and high charge mobility of the MWCNTs between the Ti and graphene sheet. Thus, we believe that our low-temperature, normal-pressure CVD process provides a simple and reproducible way to produce high quality Ti-LSG/MWCNTs for the large-scale integration of next generation graphene-based devices.

## Methods

### Experimental materials and method

Benzene was used as a carbon precursor material and was purchased from Dae-Jung Chemical and Metals Co. Ltd Korea. Titanium (IV) n-butoxide (TNB, C_16_H_36_O_4_Ti) was used as a titanium precursor and was purchased from Samchun Pure Chemical Co. Ltd., Korea. The Cu foil (99.9%) was annealed and uncoated and was used as the substrate material to grow LSG/MWCNTs. Argon and Nitrogen gas were purchased from Samchun Pure Chemical Co. Ltd., Korea. A specially-designed split Si tube furnace was divided into two parts: an inner tube and an outer tube. The dimensions of the outer tube were 30 cm length and 4 cm with two nozzles, one for the inlet of benzene vapors and the other for the inlet of Ar and N_2_ gas. The inner furnace, which is also called the heating zone, had a 10-cm length and a 5-cm diameter and was used to grow the MWCNTs/LSG on the Cu foil. Benzene was first heated below its boiling temperature to produce vapors, and these vapors were then simultaneously transferred to the tube with Ar gas, which also acts as a carrier for the benzene vapors and prevents the Cu foil from reacting with water molecules. The vapor flow and the Ar gas ratio were controlled by using control valve, and the furnace temperature increased to 500 °C in the first step, and Ar gas was released into the Si furnace. After reaching a temperature of around 500 °C, a Cu foil was inserted in the inner tube, followed by a controlled amount of Ar gas and benzene vapors. The growth of the MWCNTs/LSG was verified at different reaction times (1, 2.5, 4, and 5 minutes) at 500 °C, and the samples that were obtained were labeled as G1, G2, G3 and G4, respectively.

### Attachment of TiO_2_ on the graphene sheet

The large area graphene that was obtained was further decorated with TiO_2_ nanoparticles under the same conditions. The TNB precursor material was heated with a magnetic churn stirrer to produce TNB vapors at 100 °C. Nitrogen gas was used to carry the TNB vapors inside of the inner reaction chamber of the CVD, and the temperature was kept constant at 500 °C for 5 minutes. We found sheet-type TiO_2_ attached to the graphene sheet, and the growth of the TiO_2_ resembles the shape of the MWCNTs and was initially produced at a longer reaction time. In the case of the MWCNTs, the island that was found on the surface of the Cu sheet at a temperature of 500 °C provided the seed for the MWCNTs growth. After controlling the temperature and the reaction time, the seeds spread in different directions to form a graphene sheet.

### Instrumentation

A Raman analysis was carried out to determine the signature of graphene on the metallic substrate. The measurement was performed using a “labRam Aramis” Horiba Jobin Yvon spectrometer. A 514-nm argon-ion laser was used for the measurements, and the measurements were performed using a backscattering geometry. The spot size of the Raman excitation beam is about 1 μm in diameter. A field emission scanning electron microscope (FESEM (JSM)-5200 JOEL, Japan) was used because it offers expanded imaging capabilities and is customizable in terms of the performance requirements for the analysis. High-resolution transmission electron microscopy (HRTEM) JEOL, JEM-2012, Japan was used to determine the state and the sheet morphology of the prepared graphene. A transmission electron microscope (TEM) at an acceleration voltage of 200 kV was used to investigate the number and the stacking state of the graphene layers on the various samples. Atomic force microscopy (AFM) was then carried out using Park NX 20, Korea.

## Additional Information

**How to cite this article**: Ullah, K. and Oh, W.-C. Fabrication of large size graphene and Ti- MWCNTs/ large size graphene composites: their photocatalytic properties and potential application. *Sci. Rep.* **5**, 14242; doi: 10.1038/srep14242 (2015).

## Supplementary Material

Supplementary Information
