# Supplementary material for: Fabrication of large size graphene and Ti- MWCNTs/ large size graphene composites: their photocatalytic properties and potential application
Source: Sci Rep. 2015 Sep 18;5:14242. doi: 10.1038/srep14242 (PMC4585562; doi:10.1038/srep14242)
Supplement: Supplementary Information [file srep14242-s1.doc]

**SUPPLEMENTARY INFORMATION**

**Fabrication of large size graphene and Ti- MWCNTs/ large size graphene composites: their photocatalytic properties and potential application**

Kefayat Ullah and Won-Chun Oh [[1]](#footnote-2)*

*Department of Advanced Materials Science & Engineering, Hanseo University,*

*Seosan-si, Chungnam-do, Korea, 356-706*


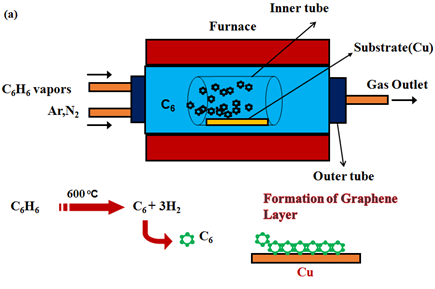


**Fig. S1** (a) Schematic design of CVD system for LSG and MWCNTs/LSG composites.

**
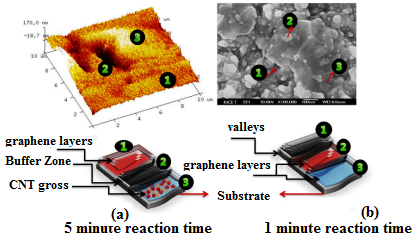
**

**Fig. S2.** The MWCNTs/LSG growth mechanism with AFM and FESEM (a-b); (a) formations and growth of MMCNTs (b) graphene formation and growth.


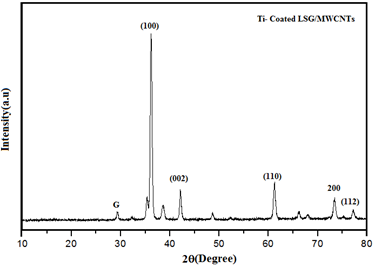


**Fig. S3** (a). XRD pattern of Ti-coated MWCNTs/LSG using CVD techniques.


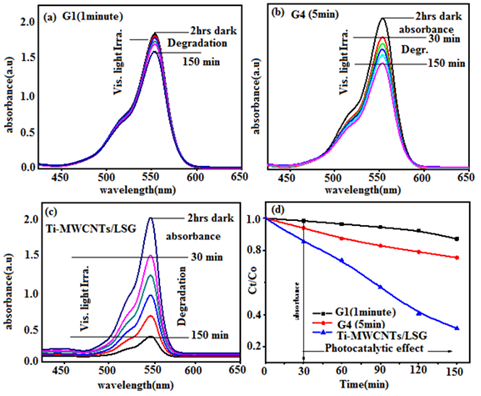


**Fig. S4.** UV-vis absorption spectra for the Rh.B degradation (a) G1 (b) G4 (c) Ti-MWCNTs/LSG (d) normalize temporal concentration vs irradiation time.

**
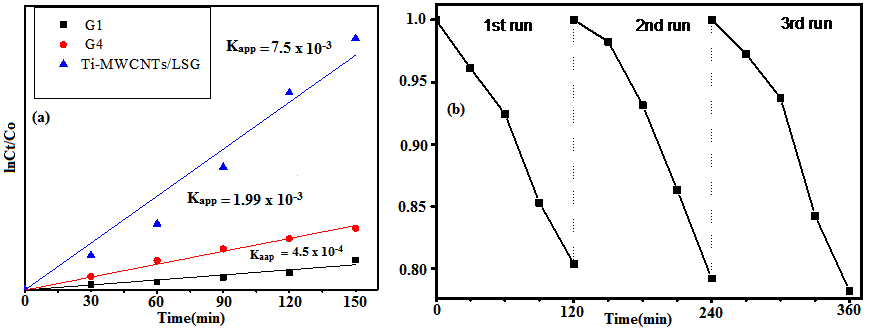
**

**Fig. S5**.(a) Apparent first order kinetics of Rh.B degradation over G1, G4 and Ti-MWCNTs/LSG nanocomposites under visible light (b) cyclic test of Ti-MWCNTs/LSG.

**
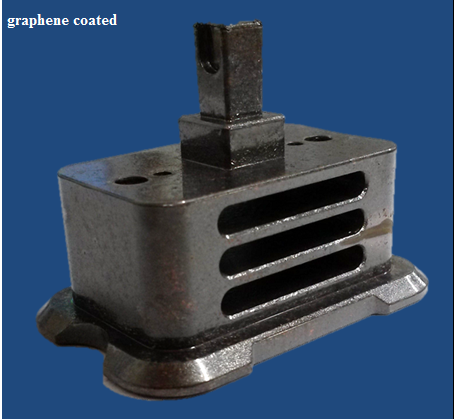
**

**Fig. S6.** Coating of LSG on semiconductor index device using our CVD techniques.

1. ^^ Corresponding author

   E-mail: [wc_oh@hanseo.ac.kr](mailto:wc_oh@hanseo.ac.kr)

   Tel: +82-41-660-1337, Fax:+82-41-688-3352 [↑](#footnote-ref-2)
